# Study Protocol: Randomised controlled trial to investigate the functional significance of marginal riboflavin status in young women in the UK (RIBOFEM)

**DOI:** 10.1186/1471-2458-9-90

**Published:** 2009-03-26

**Authors:** Marilyn HE Hill, Sohail Mushtaq, Elizabeth A Williams, Jack R Dainty, Hilary J Powers

**Affiliations:** 1 Human Nutrition Unit, School of Medicine and Biomedical Sciences, University of Sheffield, UK; 2 Institute of Food Research, Norwich, UK

## Abstract

**Background:**

The functional significance of moderate riboflavin deficiency as it is currently assessed is not well understood. Animal and human studies have suggested a role for riboflavin in the absorption and mobilisation of iron and as such may be important in maintaining haematological status. Recent National Diet and Nutrition Surveys in the United Kingdom have shown that young women in particular are at risk of moderate riboflavin deficiency and low iron status.

**Methods/Design:**

A randomised placebo controlled intervention trial was conducted to investigate the effect of riboflavin supplementation on various measures of haematological status in a group of moderately riboflavin deficient young women aged 19 to 25 years. Women who were low milk consumers were initially screened for riboflavin status as assessed by the erythrocyte glutathione reductase activation coefficient assay (EGRAC). One hundred and twenty three women with EGRAC values >1.40 were randomised to receive 2 mg, 4 mg riboflavin or placebo for 8 weeks. In addition 36 of these women were randomly allocated to an iron bioavailability study to investigate the effect of the intervention on the absorption or utilisation of iron using an established red cell incorporation technique.

**Discussion:**

One hundred and nineteen women completed the intervention study, of whom 36 completed the bioavailability arm. Compliance was 96 ± 6% (mean ± SD). The most effective recruitment strategy for this gender and age group was e-communication (e-mail and website). The results of this study will clarify the functional significance of the current biochemical deficiency threshold for riboflavin status and will inform a re-evaluation of this biochemical threshold.

**Trial Registration:**

Current Controlled Trials Registration No. ISRCTN35811298

## Background

Although National Diet and Nutrition Surveys in the United Kingdom have reported a high prevalence of poor riboflavin status in certain sections of the population, in particular adolescent girls (15–18 years) [1] and young women (19–24 years) [2], the functional significance of marginal riboflavin deficiency is not well understood.

It has been reported that 95% of adolescent girls (15–18 yr) and 75% of young women (19–24 yr) in the UK have poor riboflavin status as measured by the erythrocyte glutathione reductase activation coefficient assay (EGRAC) [1,2]. The EGRAC assay is a well-established measure of riboflavin status and reflects the availability of the FAD cofactor for glutathione reductase in red blood cells. Although EGRAC is generally considered to be a functional measure of riboflavin status, the exact relationship between EGRAC values and any specific function has not been well established. Studies in animal models have highlighted adverse effects of riboflavin deficiency on various aspects of iron handling [3,4], but supporting evidence from human studies is limited. Randomised controlled trials carried out in riboflavin deficient [5,6] and moderately deficient [7] populations have suggested that riboflavin supplementation might have beneficial effects on measures of haematological status in these groups.

Although riboflavin deficiency is highly prevalent among young women in the UK, and poor iron status, expressed as low plasma ferritin, shows a moderate prevalence (30%) [2], it is not known whether there is any causal relationship and therefore whether improving riboflavin status will have any beneficial effect on measures of iron status.

### Study Aim

The overall aim of the study was to investigate the effect of a riboflavin supplement on a comprehensive range of haematological parameters and iron bioavailability in a group of young women with low riboflavin status.

### Objectives

The specific objectives of the study were as follows: -

• To recruit women with potentially low riboflavin status based on low milk consumption

• To screen recruited women for low riboflavin status

• To randomise women with low riboflavin status to an eight week placebo-controlled riboflavin intervention trial

• To measure the effect of the intervention on measures of riboflavin status, other B vitamin status and iron status

• To measure the effect of the intervention on iron bioavailability in a subgroup of the women

• To evaluate the diets of the women recruited to the study

## Methods/Design

The study design was a randomised, double-blind, placebo-controlled, intervention trial in which participants received one of two daily doses of riboflavin or placebo over a period of eight weeks and effects on measures of iron status and iron handling were evaluated. Ethical approval for the study was granted by the Sheffield University Research Ethics Committee (SMBRER15).

### Recruitment

Recruitment started in May 2006. The sample frame consisted of healthy women, aged 19–25 yr, living in the city of Sheffield, UK. The target was to recruit 200 women to the screening part of the study, as it was estimated that 66% of the sample would have low riboflavin status, judged as an EGRAC >1.40. In order to target women with a low riboflavin status, advertisements were sent out specifically asking for individuals who consumed less than 250 ml of milk per day since milk and milk products are the major contributors to riboflavin status in young women in the UK [2].

Recruitment strategies included approaching women from an existing volunteer database held at the Human Nutrition Unit at Sheffield University, a general e-mail campaign to students and staff at both the Sheffield Hallam University and the University of Sheffield, and the use of strategically-placed posters in the local area and advertisements in the local newspaper. Leaflets were also handed out to medical students at lectures. A website was developed to provide further information and allow prospective volunteers to choose a suitable time for their first visit to a number of screening clinics held at the unit. The information sheet was also available for downloading.

Volunteers were offered financial reimbursement for their time and effort according to the level of their involvement.

### Inclusion criteria

• Female

• Consumers of less than half a pint of milk (250 ml) a day

• Healthy

• Aged 19 – 25

### Exclusion criteria

• Regular blood donors (no donations 3 months before starting or during the study)

• Regular use of multivitamins or iron supplements

• Diagnosed gastrointestinal problems such as Inflammatory Bowel Disease or Coeliac disease

• Haematological disorders

• Haematochromatosis

• Pregnant or lactating

### Screening Clinic

The purpose of the screening clinic was four fold: -

1. to establish eligibility on the basis of the inclusion and exclusion criteria

2. to provide the volunteer with further information and gain informed consent for the whole study including the bioavailability arm

3. to collect the following demographic and health information by personal interview: -

• date of birth

• contact details

• GP address

• the use of medication

• supplement use

• the presence of known food allergies

• the existence of any medical conditions

• smoking status

• menstrual information – date of last period, length of cycle and contraceptive usage

4. to collect a 500 μl, non-fasted, finger prick sample for the measurement of riboflavin status (EGRAC) and plasma ferritin concentration.

Women with an EGRAC >1.40 were considered to be moderately riboflavin deficient and were invited to take part in the intervention study. Those women accepting the invitation were randomised to receive one of three supplements; 4 mg riboflavin, 2 mg riboflavin or placebo. The capsules were formulated by Research Products Limited (RPL, Blackpool) and were suitable for vegetarians and vegans (gelatin-free and coloured with chlorophyll). The researchers and participants were blinded to the identity of each capsule; only the suppliers of the capsules and an independent third party responsible for the randomisation knew the identity. The capsules, packed in pots of 60, were given at the first baseline visit and were sufficient for the entire intervention allowing 4 spare capsules for any losses. The riboflavin content in a random sample of each type of capsule was checked by high performance liquid chromatography.

### Randomisation to treatment

Randomisation was performed by an independent third party. Treatment codes were generated by ferritin stratification in blocks of 3 using a computer generated randomisation schedule. Three ranges of plasma ferritin were used for stratification; less than 15 ng/ml, 15.1–59.9 ng/ml and greater than 60 ng/ml. One in four women were allocated to the bioavailability arm by random sampling from those in the 4 mg and placebo groups of the intervention.

### Protocol for the intervention study

All eligible women were invited to take part in the intervention study (main study). As far as possible the dates for baseline blood collections were arranged to coincide with the middle of the menstrual cycle to reduce any effects of menstrual bleeding on measures of iron status. Once dates for all clinic visits had been agreed with the volunteer, an information pack containing a 4 day food diary, food portion booklet and a guide to the study was sent by mail.

Subjects attended the Clinical Research Facility at the Royal Hallamshire Hospital, Sheffield for all study clinics, which were held between 8.00 am and 10.00 am. Women were requested to fast from midnight the previous night.

Frequent contact was maintained by telephone or by email to remind the volunteers of clinic dates, the requirement for a fasted blood sample and the need to complete food diaries.

### Details of clinics

**Figure 1:**
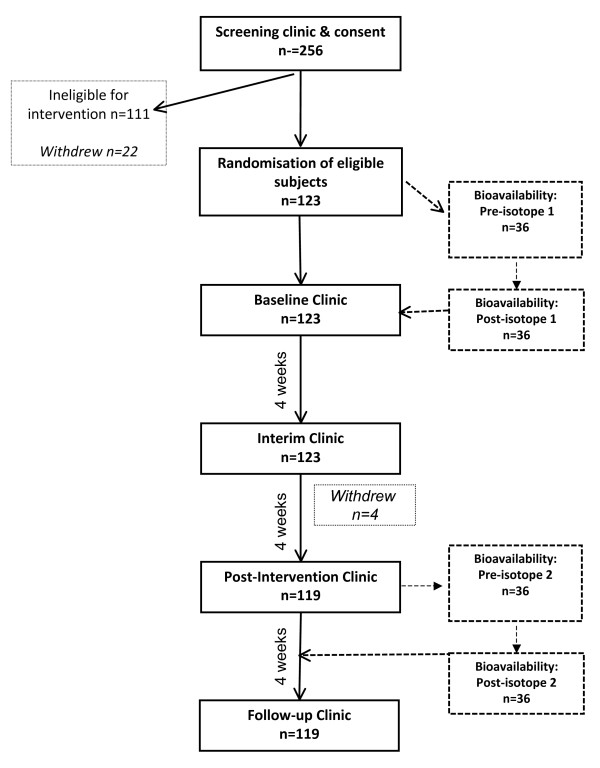
**Flow diagram for the RIBOFEM study**.

Women attended clinics according to whether they had been recruited to the intervention study alone or to the additional bioavailability study. The scheme for the clinic protocol is given in figure 1.

### Intervention study clinics

#### Baseline Clinic (Week 0)

This was an opportunity to explain the study in more detail and answer any questions. The height and weight of each subject were measured. The completed food diary was returned by the volunteer and the responses discussed with the researcher. The next food diary was given and dates agreed for completion prior to the next visit. A fasted blood samples was taken (15 ml, EDTA vacutainer) for baseline biochemistry and haematological measurements. Volunteers were given capsules with instructions for their use.

#### Interim clinic (Week 4)

A finger prick blood sample was taken (500 μl, EDTA microvette) for the measurement of EGRAC and ferritin, to allow the detection of early response to intervention and to check compliance. The second completed food diary was collected and discussed.

#### Post Intervention clinic (Week 8)

A fasted blood sample was collected (15 ml, EDTA vacutainer) for post intervention biochemistry and haematological measurements. Any remaining capsules were collected and counted.

#### Follow-up clinic (Week 12)

A further blood sample was taken (5 ml, EDTA vacutainer) for EGRAC and ferritin measurements to allow evaluation of the persistence of any response to intervention.

### Additional Bioavailability Clinics

A sub-sample (one in four) of the women eligible for the main intervention study was chosen at random from the placebo and 4 mg groups to take part in the bioavailability study, in addition to taking part in the main study. Further information regarding this aspect of the study, including food diaries and food portion booklets, was sent to the women prior to attending the first clinic.

In particular they were advised not to eat red meat for 24 hours or drink alcohol for 12 hours prior to the first clinic. They were also advised not to take part in any vigorous exercise for the 24 hours before the clinic.

Women on the bioavailability arm were required to attend for extra clinics and extra procedures as described below (see fig. 1).

#### Bioavailability: first pre-isotope clinic (2 weeks before the intervention study)

At this initial clinic, fasted volunteers attended the Clinical Research Facility (CRF) to receive the initial dose of the stable isotope labelled iron (^58^Fe = 95.1%). Prior to receiving this first dose, 5 ml blood were collected into a trace element free lithium heparin vacutainer and transferred into acid washed microcentrifuge tubes for background isotopic analysis. The labelled iron was given in two separate 2 mg doses, one with a specially prepared breakfast and the other with a specially prepared lunch. The isotope was prepared in aqueous solution (2 mg Fe as a ferrous sulphate solution per tube) by the Institute of Food Research (Norwich) and transported to Sheffield on dry ice. The aliquots of isotope were stored at -20°C until required. Just prior to administration each dose was diluted with a lemonade drink and given to the volunteer within 10 minutes of preparation. The drink containing the labelled iron was given with a simple breakfast of toast with jam and dairy-free spread, consumed at 0900 h. Four hours later the volunteers were given a lunch of pasta and instant tomato sauce, a plain bread roll and a second drink containing the 2 mg dose of labelled iron. Beverages and foods were carefully chosen so as not to contain any known enhancers or inhibitors of iron absorption. The beverage was free from phosphoric acid, tannin and vitamin C according to the manufacturers product description and the flavour and fizzy nature of the drink masked any taste of the iron. Additionally meals were chosen on the basis of a negligible iron or riboflavin content. The pasta sauce was selected on the basis of negligible vitamin C content and this was confirmed by enzymatic analysis using the method described by Vuilleumier [8], (< 1 mg/meal), using an automated centrifugal analyser (COBAS BIO Roche Products). Volunteers were allowed to leave the facility between meals but instructed not to eat, drink or exercise between the meals or for two hours after the lunch. Water was permitted ad libitum.

This clinic was also an opportunity to explain the study in more detail to the bioavailability volunteers, to answer any questions and to record the height and weight of each subject. The food diary was collected and the responses discussed with the researcher. The next food diary was given and dates agreed for completion prior to the next visit.

#### Bioavailability: first post-isotope clinic

This clinic coincided with the baseline visit for the intervention study. In addition to the blood samples taken for the intervention study afurther fasted blood sample (5 ml, trace element free heparin vacutainer) was taken for the bioavailability study.

#### Bioavailability: second pre-isotope clinic

This clinic coincided with the post intervention clinic of the main study. At this clinic women repeated the labelled iron, meal, and blood sampling regimen described above as well as the blood sampling regimen for the intervention study.

#### Bioavailability: second post isotope clinic (2 weeks after the end of the intervention)

Fasted blood was collected (5 ml, trace element free lithium heparin vacutainer) and transferred into acid washed tubes for the post riboflavin intervention component of the bioavailability study.

### Compliance

The capsules remaining at the end of the study were collected and counted and used as a measure of compliance.

### Power Calculation

There is a lack of consistency in the design of studies that report an improvement in haemoglobin following riboflavin supplementation, in terms of size of dose and duration of intervention and baseline riboflavin and iron status. However, Buzina's study [7] is the most relevant in that the study population was only moderately riboflavin deficient and less so than the subjects we anticipated recruiting. This group showed an increase of 0.7 g/dl Hb over 12 weeks of a modest supplement of 3 mg riboflavin per day.

### Details

We calculated that to achieve a 0.7 g/dl improvement in Hb in a population with an estimated baseline Hb of 13.5 g/dl (SD 1.05) [2] we required 31 subjects per arm of the study. This would give a power of 90%. We aimed to recruit 40 volunteers to each arm, allowing close to 15% dropout.

Based on an expected level of deficiency in this population of 66% it was anticipated that we would need to screen at least 200 young women.

#### Power calculation for the bioavailability

It was estimated that 15 subjects per group would be required to detect a 5% difference between the 4 mg and placebo intervention groups in iron incorporation into erythrocytes in response to intervention. This estimate was based on a mean bioavailabilty of 15% and a SD of 5% [9]with a power of 90% and a significance level of 0.05.

### Dietary Analysis

To evaluate any dietary changes during the intervention, volunteers were asked to complete a four-day food diary before the baseline visit and again before the interim visit. The four days included one weekend day. The diary was sent to the participant prior to the first visit and then discussed with the researcher at the first visit for checking legibility, correct entry of food types and portion sizes. The second diary was given at this visit for completion prior to the next interim visit and the instructions explained again if necessary.

The diaries were developed by the Institute of Food Research, Norwich and modified to include sufficiently detailed instructions as to make them self-explanatory. Food portion booklets based on the former Ministry of Agriculture, Fisheries and Food (MAFF) food atlas [10] were also provided to assist with estimating portion sizes. The data from the food diaries were analysed for micronutrient and macronutrient content using Windiets Research software (Robert Gordon University, UK).

### Blood collection and laboratory analysis

Blood was collected for the screening and interim visits by finger prick sampling (500 μl).

Immediately following the clinics, blood samples were taken to the laboratory, separated by centrifugation and the red cells washed with PBS and lysed with an equal volume of water. Haemolysates were stored at -20°C for the assessment of riboflavin status using the EGRAC assay. Plasma was stored at -20°C for ferritin analysis.

At the baseline and post intervention visits, blood was collected by venupuncture into vacutainers. A 10 ml blood sample was collected into EDTA tubes and separated by centrifugation into plasma and red cells. The red cells were washed and haemolysed for EGRAC measurement as before and the plasma divided into aliquots and stored at -80°C for the measurement of other variables. A further 5 ml blood were collected into a separate EDTA tube and sent to the Haematology Department at the Royal Hallamshire Hospital for full blood count and zinc protoporphyrin (ZPP) measurements.

At the follow up visit a single 5 ml sample was collected into EDTA tubes for EGRAC and plasma ferritin measurements.

For the bioavailability arm of the study a further 5 ml blood were collected at the times described in the bioavailability protocol, into heparin trace-element free tubes and the whole blood aliquoted into 4 acid washed microfuge tubes for sending to the Institute of Food Research, Norwich for isotopic analysis.

### Outcome Variables

#### Primary Outcome Variables

To assess haematological parameters the following were measured in the haematology laboratory at the Royal Hallamshire Hospital, Sheffield: red blood cell count, PCV, MCV, MCHC, haemoglobin and ZPP. ELISA kits were used to measure plasma ferritin (Spectro Ferritin Kit, ATi Atlas Ltd. Chichester, UK) and plasma soluble transferrin receptor (Quantikine DTFR1 – Human sTfR Immunoassay, R&D Systems, Abingdon, UK)

Riboflavin status was measured by the EGRAC assay [5] and plasma flavin concentration measured using an HPLC kit method (Chromsystems, Munich, Germany).

#### Secondary Outcome Variables

To adjust for any confounding effects of changes in the intake of Vitamins C, B2, B6, B12 or folate that might affect haematological status measurements were made of these nutrients. Plasma vitamin C was measured by a fluorometric assay using a COBAS Bio centrifugal analyser [8]. Plasma pyridoxal phosphate and pyridoxic acid, as measures of vitamin B6 status, were measured using an HPLC kit (Chromsystems, Munich, Germany). The routine clinical chemistry laboratory at the Royal Hallamshire Hospital measured plasma vitamin B12, and plasma folate was measured as 5-methyltetrahydrofolate by isocratic reverse phase HPLC [11].

Because of the documented influence that riboflavin status can have on plasma homocysteine concentration [12,13] plasma homocysteine was measured as an additional functional measure of riboflavin status using an iMX autoanalyser (Abbott Diagnostics, Maidenhead, Berks, UK).

In order to correct for increases in plasma ferritin as a result of underlying infection, plasma C-reactive protein was measured using an ELISA kit (Quantikine DCRP00 Human C-Reactive Protein Immunoassay, R&D Systems, Abingdon, UK).

### Data Analysis

For descriptive statistics baseline values will be analysed according to randomisation group. Data will be tested for normality using Shapiro-Wilk and Kolmogorov-Smirnov tests. Categorical variables will be tested using the Chi squared test and continuous variables compared across randomisation groups using ANOVA.

The effects of intervention on all continuous variables will be analysed by ANOVA. Where ANOVA indicates a significant difference across the randomisation groups a Scheffe test or Kruskal-Wallis Multiple Comparison Z-value test will be carried out, as appropriate, to identify where the differences lie.

Spearman Rank Correlations will be conducted to examine possible associations between selected continuous variables.

### Bioavailability data analysis

Statistical analysis will be performed at IFR using R data analysis software [14]. Standard Linear Regression and ANOVA models will be used. For all models, regression diagnostics will be checked to determine if data transformations, outlier omissions or alternative non-parametric models are required. Results will be considered significant if P < 0.05 for all tests.

### Initial Findings

#### Recruitment

Over a period of fifteen months, 256 women were screened for moderate riboflavin deficiency. From this initial screening, 145 were eligible for the intervention trial on the basis of an EGRAC>1.40. This represented 57% of our screened population. Of these 123 women agreed to take part in the main study (36 of these were randomly allocated to the bioavailability arm). In all, 4 women failed to complete the main study (none of whom, were in the bioavailability arm).

#### Compliance

The mean compliance (± SD)of the volunteers as judged by the number of capsules returned was 96 ± 6%. (range 70–100)

#### Influence of recruitment to screening on riboflavin status

It was considered possible that the time taken between screening and starting the intervention trial might influence dietary change prior to starting the intervention. The median time that women had to wait before starting the intervention was 47 days (range 7–240). The main reason for delay was the coordination of the start of the study with the menstrual cycle. Other factors included the timing of vacations and the need to accommodate 4–6 visits over a period of 12–14 weeks depending on the arm of the study.

The mean EGRAC value for the entire screened population (n = 256) was 1.43 ± 0.18.

This is comparable with the EGRAC values reported in the most recent Diet and Nutrition Survey of adults in the UK for this age group (mean, 1.45 ± 0.19).

The mean (± SD) EGRAC value of these women recruited to the intervention study was 1.54 ± 0.14. The mean EGRAC value at baseline was significantly greater (1.59 ± 0.19) indicating a modest but significant deterioration in riboflavin status during the waiting period (*P *= 0.007)

## Discussion

Recruitment to the screening and intervention stages of this study was successfully completed within 16 months. The recruitment strategies that proved to be the most effective for this age group were the use of email and a web site, probably because these are familiar media and in use daily by this age group. There was also a good response from posters and leaflets but contact was generally then made via the web site and email. A short newspaper campaign was not successful in recruiting volunteers. Constant contact by email and the frequency of the visits contributed to a low drop out rate and helped maintain a high compliance rate. Financial reimbursement was also likely to have been a significant factor in obtaining a high recruitment rate and maintaining involvement in the study.

It was thought that volunteers enrolled into a nutrition study might modify their diet so as to increase their riboflavin intake during the period from screening until the start of the intervention study. However the volunteers in this study showed a deterioration in riboflavin status, evidenced by a small but significant increase in the mean EGRAC value.

The results of this study will provide valuable information about the effect of a riboflavin supplement on the haematological status of young women with marginal riboflavin status and help us to understand the functional significance of riboflavin deficiency. If an effect is demonstrated it will form the basis of a discussion to re-evaluate the EGRAC threshold for riboflavin deficiency.

The randomised controlled trial is now complete and the statistical analysis underway.

## Competing interests

The authors declare that they have no competing interests.

## Authors' contributions

HJP and EAW were responsible for the study concept and design. MHEH and SM conducted the study. JD was responsible for the provision of the ^58^Fe isotope and erythrocyte iron incorporation analysis. MHEH drafted the manuscript and HJP provided critical revision of the final manuscript.

All authors have read and approved the final manuscript.

## Pre-publication history

The pre-publication history for this paper can be accessed here:


